# Research on pollen allergy in Fuzhou, China: from the online public concern surveys to pollen monitoring

**DOI:** 10.3389/fpubh.2026.1732606

**Published:** 2026-04-07

**Authors:** Xin Yue, Mingyong Guo, Ting Chen, Chaowu Li, Yaocheng Wang, Yaqi Liu, Hao Zheng, Zhiqiang He

**Affiliations:** 1 Department of Otolaryngology, Head and Neck Surgery, Fuzhou University Affiliated Provincial Hospital, Fuzhou, China; 2 Shengli Clinical Medical College, Fujian Medical University, Fuzhou, China; 3 Department of Clinical Laboratory, Fuzhou University Affiliated Provincial Hospital, Fuzhou, China

**Keywords:** allergic rhinitis, baidu index, epidemiology, online search interest, pollen monitoring

## Abstract

**Background:**

The role of pollen as a major aeroallergen in seasonal allergic rhinitis remains understudied in Fuzhou, representing a significant gap in the local public health literature.

**Objectives:**

This study aimed to analyze the epidemiological characteristics of pollen allergy in Fuzhou, China, by integrating local pollen monitoring data, allergic rhinitis visits with Baidu Index search trends from both Fuzhou and the national level for comparative analysis.

**Methods:**

This study analyzed search data from Baidu Index, pollen monitoring and allergic rhinitis visits data in Fuzhou (2023.6–2025.6). Statistical analyses were performed using R and Excel (*p* < 0.05).

**Results:**

Baidu Index data showed distinct seasonal patterns: “pollen allergy” search volumes exhibited bimodal peaks nationwide, while “allergic rhinitis” search volumes displayed broad peak (March-October) in Fuzhou. Statistically significant correlations between the keywords were found in the national level (China: *r* = 0.655, *p* < 0.001, Fuzhou: *r* = −0.07, *p* = 0.741). Two-year pollen monitoring in Fuzhou recorded a total concentration of 43,023 grains/m3, with significantly higher levels in the first year (*p* < 0.001). Clear seasonal peaks were observed in spring (tree pollen: Moraceae, Pinaceae, Cupressaceae) and autumn (grass pollen: Urticaceae, Poaceae). Five dominant families accounted for 66.5% of total pollen. The pollen concentration showed a significant positive correlation with the keyword “pollen allergy” (*r* = 0.649, *p* < 0.001); the correlation between pollen concentration and “allergic rhinitis” did not reach statistical significance (*r* = 0.242, *p* = 0.244). The total number of allergic rhinitis visits reached 42,166, the correlation analysis revealed no statistically significant association between daily allergic rhinitis visits and pollen concentration levels (*r* = −0.071, *p* = 0.056).

**Conclusions:**

By integrating pollen monitoring data with online search data (Baidu Index) and allergic rhinitis visits, this study provides insights into pollen exposure in Fuzhou and its relationship with public health countermeasures.

## Introduction

The “hygiene hypothesis” suggests that as living standards improve and hygiene practices become more emphasized, the prevalence of infectious diseases decreases while immunologic diseases increases (1).

Allergic rhinitis (AR) is an immunologic condition, with high prevalence rates reported both internationally and across China (2–5). A major trigger of seasonal AR is airborne pollen. According to a multicenter study conducted across 13 cities in China, the peak incidence of seasonal allergic rhinitis in China occurs during spring and autumn, showing a close correlation with airborne pollen concentrations (6).

Pollen contains a variety of allergenic proteins, with more than 150 identified as capable of inducing allergic sensitization (7). Upon initial exposure, patients develop specific IgE antibodies, subsequent re-exposure to these substances triggers mast cell degranulation, releasing bioactive substances that cause varying degrees of allergic reactions in patients (3, 8). Pollen grains range in diameter from 5 to 200 μm, and thus typically settle in the upper respiratory tract when inhaled. Meanwhile, studies have shown that nasal symptoms are the most prominent and frequently reported manifestations among affected individuals (9).

An allergic reaction occurs when a patient inhales a quantity of sensitizing pollen exceeding the threshold concentration (9). This pollen concentration threshold is influenced by an individual's genetic background, the pollen type, and environmental factors (10, 11). During pollen dispersal seasons, the discomfort experienced by pollen allergy sufferers severely impacts their daily lives, work, and studies, while also imposing medical and economic burdens (12). Therefore, studying the distribution and dispersal patterns of airborne pollen in the region can provide important information for the prevention and treatment of pollen allergies.

Due to variations in regional vegetation, topography, climate conditions, pollution levels, and human factors, pollen concentrations can exhibit significant differences across geographical areas and seasonal periods (13–15).

Pollen monitoring serves as an essential source of objective environmental allergen exposure data. The two primary techniques employed are gravitational settling and volumetric sampling. Gravitational methods, such as the Durham sampler, use adhesive-coated slides placed on an elevated platform, where pollen particles settle passively by gravity. Results are reported as particles per unit area. While simple and low-cost, this approach is limited by reduced deposition under high wind conditions and susceptibility to dust contamination. In contrast, volumetric sampling, exemplified by the Hirst-type Burkard volumetric spore trap, actively draws air at a controlled rate, capturing particles on an adhesive tape or drum. This system allows continuous operation for up to seven days, significantly reducing operational burden. In the Burkard, air is drawn into a 14 mm x 2 mm orifice at 10 liter per minute, and any airborne particles with sufficient inertia are impacted on either a greased tape or a greased microscope slide beneath the orifice. The impaction surface moves past the orifice at 2 mm per hour permitting time-discriminate analysis. There is also a wind vane attached to the sampler head; since the head is able to rotate the orifice is always oriented into the wind. Data are expressed as allergen concentration per unit volume, offering a more accurate reflection of real-world atmospheric conditions. The Burkard sampler also shows high efficiency in collecting fine particles, including pollen and mold spores under 10 μm (16, 17).

Conventional ground-level pollen monitoring has long provided valuable objective data on environmental allergen exposure (18, 19). In this study, pollen monitoring was conducted using a Hirst-type Burkard volumetric spore trap situated at a representative urban site in Fuzhou.

However, this approach faces limitations due to sparse station coverage, substantial operational costs, and logistical constraints, making it difficult to accurately characterize population-wide exposure.

In recent years, it has been shown that using Internet data is an effective complement to traditional surveillance methods with the advantages of high real-time availability, rapidity, and low cost (20). Estimates using Google trends by American researchers were consistently 1–2 weeks ahead of traditional surveillance systems reports (21); hay fever detection from Twitter by Australian researchers can guide the prevention of seasonal AR as well as medications (22). This is essential for the timely and effective development of prevention policies.

China has also accumulated a huge amount of Internet data, and how to use it to assist clinical disease analysis has become an important issue worth studying. Currently, Baidu dominates China's search engine market, offering vast data storage and research value. The application of Baidu Index in studying public responses to diseases demonstrates both scientific validity and practical utility. Many studies have shown that the frequency of queries for Baidu keywords for allergy diseases and related symptoms is closely related to the patient's symptoms, which can reflect the actual demand trend of searchers (23–26). Internet data provides a real time, low cost, and wide coverage behavioral data source for allergy research, which is particularly suitable for tracking disease trends, assessing public health needs, and optimizing prevention and control strategies.

Fuzhou, the capital of Fujian Province, is located in southern China, west of the Taiwan Strait, between latitudes 25°15′-26°39′N and longitudes 118°08′-120°31′E. Despite the high prevalence of allergic rhinitis nationwide, the specific drivers and patterns of pollen allergies in Fuzhou have not been adequately investigated in recent years. According to the Fuzhou Forestry Bureau (https://fzly.fuzhou.gov.cn/lygk/), the city has a forest area of 729,400 hectares, with a forest coverage rate of 51.77%, consistently ranking among the highest of all provincial capital cities in China. Its predominant vegetation type is coniferous forest, mong these, Masson pine (*Pinus massoniana* Lamb.) and Chinese fir [*Cunninghamia lanceolata* (Lamb.) Hook.] are the most extensively distributed. A recent local survey identified 52 species of allergenic plants across 23 urban parks in Fuzhou, accounting for 9.11% of the total plant species investigated, and the dominant allergenic trees included conifers, parkas and sequoias, while major allergenic grasses comprised dogbane, matang and knotweed (27). Meanwhile, as reported by the Fuzhou Municipal Government, Fuzhou features a typical subtropical monsoon climate, marked by long summers, short winters, and over 300 frost-free days per year. Influenced by the East Asian monsoon, the weather exhibits variability and is prone to extremes. These characteristics amplified the impact of environmental factors, contributing to the complex morbidity patterns of diseases such as allergies in this region. There is an urgent need for updated studies that examine how local pollen dynamics, influenced by Fuzhou's distinctive subtropical monsoon climate, contribute to the onset and severity of seasonal allergy symptoms.

Based on the above background, we planned to explore the epidemiological characteristics of pollen allergy in Fuzhou by using internet search data (Baidu Index), pollen monitoring and outpatient allergic rhinitis visits data.

## Methods

### Baidu index keywords selection

This study mainly analyzed the temporal search trends of pollen allergy-related terms in Fuzhou. The Internet search data was sourced from the Baidu Index platform. Searches were conducted at two geographical levels: Fuzhou City and the entirety of China. And the daily search volume of the above terms was covering the period from June 1, 2023, to June 30, 2025. These following keywords were selected: “pollen allergy” and “allergic rhinitis.”

### Airborne pollen data

**Equipment:** Airborne pollen monitoring was conducted using a 7-day, 24-h Hirst-type Spore Trap (Burkard, UK). **Sampling Site:** The pollen monitoring site was established in Gulou District, Fuzhou City, Fujian Province. The sampler was placed on an outdoor platform on the fifth floor of a building (Latitude 26°09′N, Longitude 119°30′E), approximately 18 meters above ground level. The location was characterized by unobstructed air circulation, no overhanging vegetation in close proximity, and no surrounding buildings causing sheltering effects. A stable long-term power supply was available. **Sampling Period:** Pollen sampling was carried out from June 8, 2023, to June 7, 2025, covering a total period of two years. **Slide Preparation:** After each sampling cycle, the adhesive tape (total length 336 mm) was removed from the drum and cut into segments of 48 mm, each representing a 24-h sampling period. Seven glass slides were prepared for each week. Dates were labeled accordingly, and glycerin gelatin mounting medium containing basic fuchsin stain was applied to fix and stain the pollen grains. **Pollen Concentration Calculation:** Daily pollen counts were obtained by microscopically examining under a light microscope at 400 × magnification. Pollen concentration was calculated using the standardized volumetric method with the following formula: C = N × W / (F × V), where: C: pollen concentration (grains/m3), N: pollen count under microscopy (counted over a 48 mm segment of the tape), W: width of the sampling tape (14 mm), F: diameter of the microscope objective field (0.55 mm), V: total air volume sampled over 24 h; with a flow rate of 10 L/min, the daily total was 14.4 m3; as recommended, three longitudinal transects were performed in this study to obtain a representative estimate, therefore, pollen grains per cubic meter of air = N × 0.59 (28). The annual cumulative pollen exposure indicator is represented using the annual pollen integral (APIn) (29). **Pollen Identification:** Pollen grains identifiable to the family level. Another Pollen types with very low concentration, unidentifiable grains, or pollen fragments were only documented by concentration without further taxonomic detail.

### Allergic rhinitis visits data

Daily outpatient numbers for allergic rhinitis consultations between June 8, 2023, and June 7, 2025, were obtained from the Hospital Information Center of Fuzhou University Affiliated Provincial Hospital. These data encompass all diagnosis-coded outpatient visits and exclude emergency department records.

### Statistical analysis

R (4.3.2 version) and Microsoft Excel were used in this study. Shapiro-Wilk test was used to verify the normality of data sets. For data following a normal distribution, the Pearson correlation is used for correlation analysis, and the independent samples *T*-test is applied for difference analysis. For data that do not follow a normal distribution, the Spearman correlation is employed for correlation analysis, and the Wilcoxon Signed-Rank Test is used for difference analysis. Statistical significance was set at *p* < 0.05.

## Results

### Description of the baidu keyword search trends

**Figure 1:**
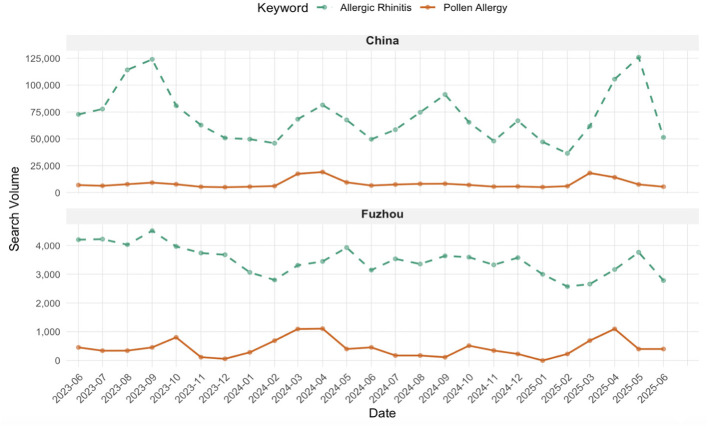
Baidu index search trends in Fuzhou and China from June 2023 to June 2025.

From June 2023 to June 2025, significant public interest in allergies was maintained, characterized by periodic shifts corresponding to seasonal changes (Figure 1). The total Baidu Index search volumes are as follows: the allergens-related keywords “pollen allergy” (China: 212,675, Fuzhou: 10,999); the disease-related keywords “allergic rhinitis” (China: 1,778,588, Fuzhou: 87,012).

**Figure 2:**
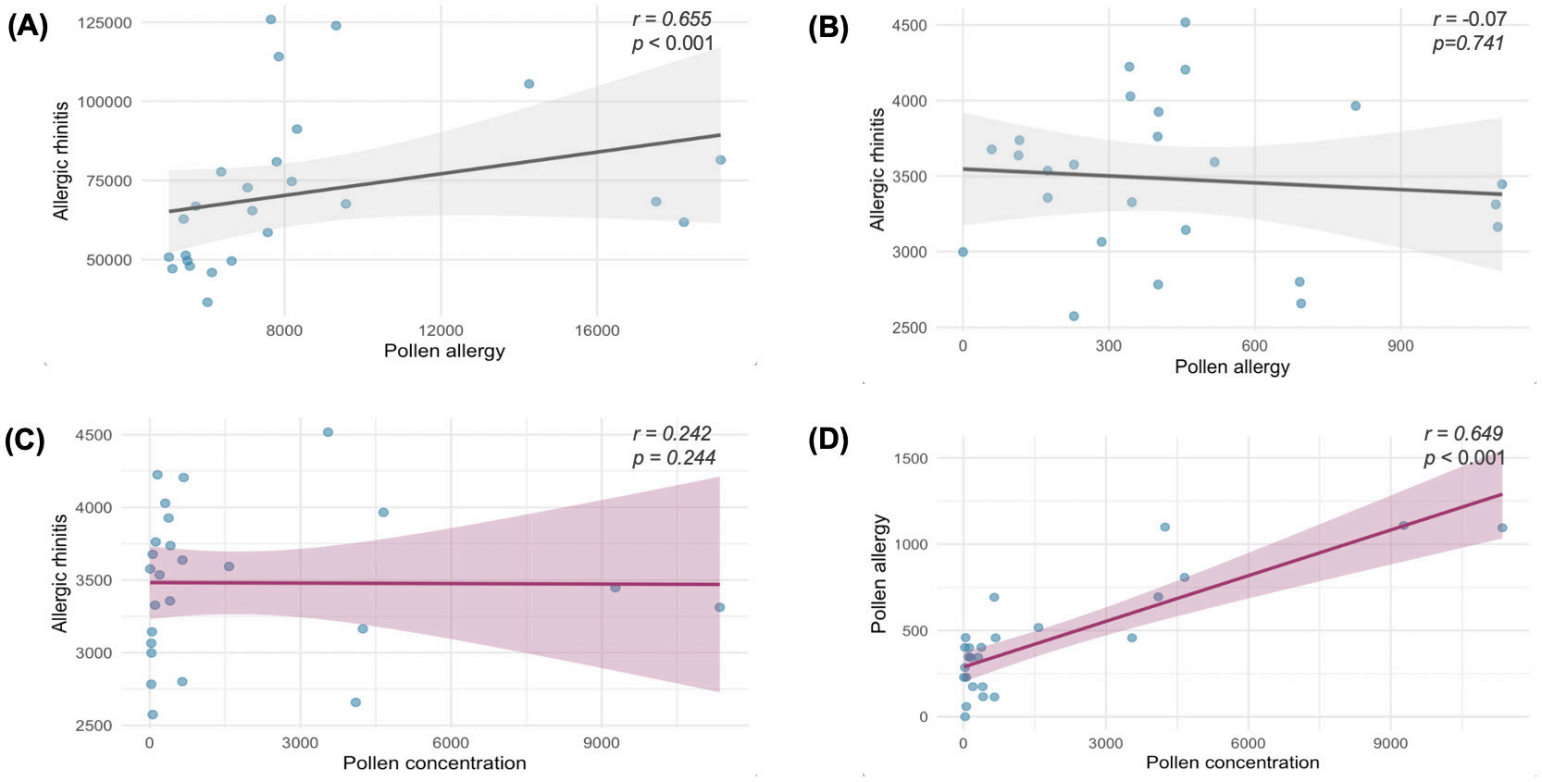
Correlation analysis of Baidu index keywords and pollen concentration [**(A)** Correlation between keyword “pollen allergy” and “allergic rhinitis” at the national level; **(B)** Correlation between keyword “pollen allergy” and “allergic rhinitis” at the Fuzhou level; **(C)** Correlation between pollen concentration and keyword “allergic rhinitis” in Fuzhou; **(D)** Correlation between pollen concentration and keyword “pollen allergy” in Fuzhou].

Analysis of monthly trends revealed distinct patterns. In Fuzhou, searches for “pollen allergy” showed two annual peaks in March–April and September–October, while searches for “allergic rhinitis” exhibited minor corresponding increases during these months, overall forming a broad sustained peak from March to October. In contrast, at the national level, searches for “pollen allergy” displayed two prominent annual peaks in March–May and August–November, and searches for “allergic rhinitis” showed clear corresponding peaks with more pronounced seasonal fluctuations. Spearman correlation analysis (Figures 2A, B) indicated a statistically significant correlation between the two search terms at the national level (*r* = 0.655, *p* < 0.001). In contrast, no significant correlation was observed in Fuzhou (*r* = −0.07, *p* = 0.741).

### Pollen monitoring in Fuzhou

**Figure 3:**
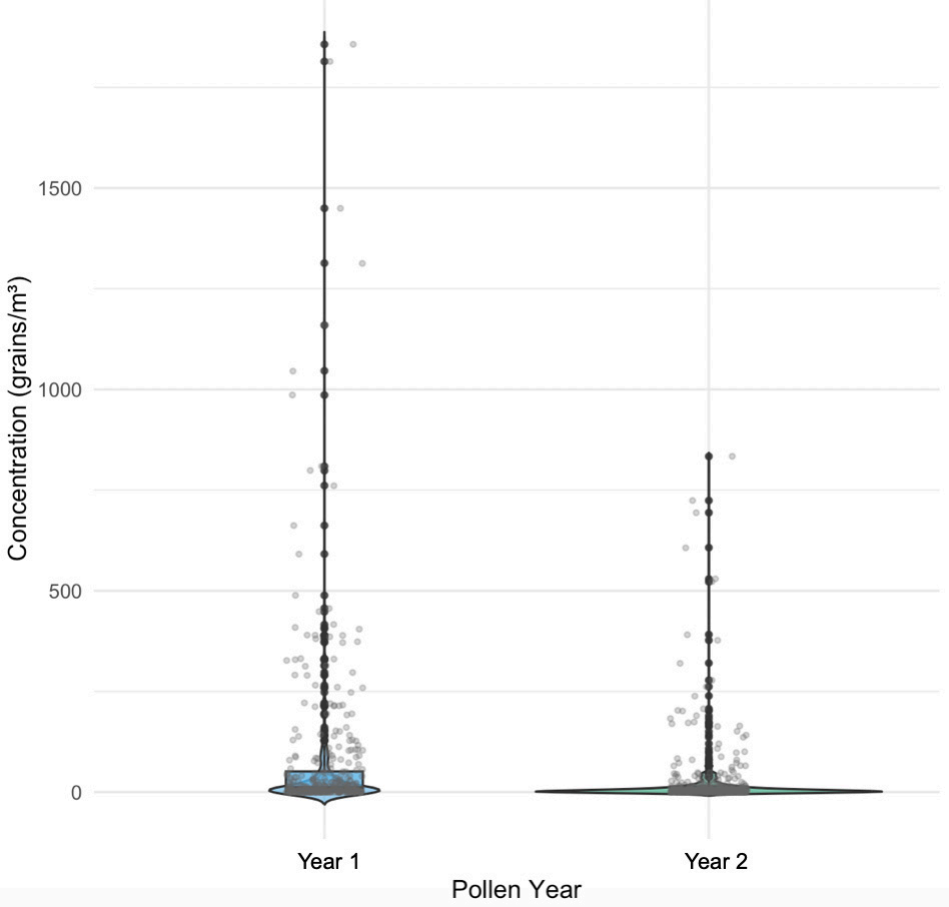
Comparison of pollen concentrations between the first and the second year.

Over the two-year monitoring period (731 days), pollen was detected on 517 days, accounting for 70.7% of the observation period. The daily pollen concentration ranged from 2 to 1,857 grains/m3. The total pollen concentration collected at the monitoring site was 43,023 grains/m3. The APIn in the first year was 31,479 grains/m3, compared to 11,544 grains/m3 in the second year. Shapiro-Wilk tests indicated that daily pollen concentrations did not follow a normal distribution. Subsequent Wilcoxon signed-rank test revealed that the pollen concentration in the first year was significantly higher than that in the second year (*Z* = −7.581, *p* < 0.001) (Figure 3). The median pollen concentration in the first year was 9 grains/m3, significantly higher than 4 grains/m3 in the second year. The first year exhibited a wider data distribution with an interquartile range (IQR) of 2-52 grains/m3 (IQR = 50) and contained numerous high-concentration outliers, reaching a maximum of 1,857 grains/m3. In contrast, the second year showed a more concentrated distribution (IQR = 14) with generally lower concentration levels.

**Figure 4:**
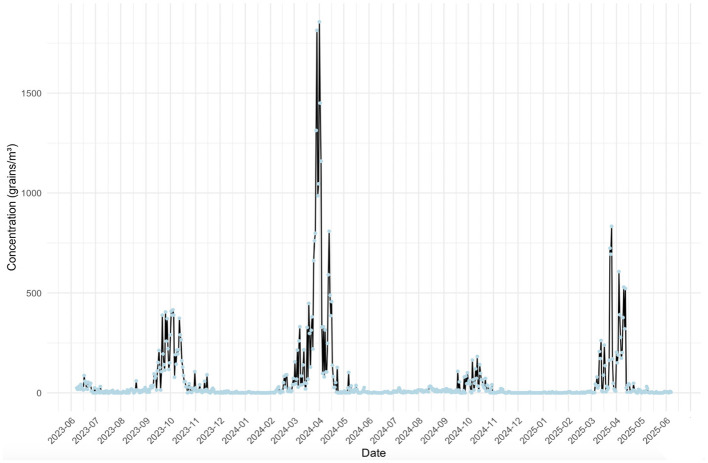
Time series of daily pollen concentration in Fuzhou from June 2023 to June 2025.

Time series of daily pollen concentration in Fuzhou from June 2023 to June 2025 was showed in Figure 4. The pollen monitoring data revealed two distinct dispersal peaks each year, with the spring peak being more prominent than the autumn peak. The primary peak occurred from March to April, dominated by tree pollen such as Moraceae, Pinaceae, and Cupressaceae. In the first year, the total concentration during this season reached 20,621 grains/m3, accounting for 65.5% of the annual total. The peak daily concentration was recorded on April 1 at 1,857 grains/m3. In the second year, the spring season total was 8,344 grains/m3, representing 72.3% of the annual total, with a peak concentration of 834 grains/m3 observed on March 26. A secondary peak was observed from September to October, primarily consisting of grass pollen including Urticaceae and Poaceae. In the first year, this autumn period contributed 8,202 grains/m3, comprising 26.1% of the annual total. The peak daily concentration during this season was 416 grains/m3 on October 4. In the second year, the autumn season total was 2,227 grains/m3, accounting for 19.3% of the annual total, with a peak of 182 grains/m3 recorded on October 12. During May to August of both years, limited amounts of tree and grass pollen were detected. From November to February of the following year, pollen levels were minimal, representing a relative pollen intermittency period.

The recorded pollen grains belonged to 15 families, including Amaranthaceae, Betulaceae, Casuarinaceae, Compositae, Cupressaceae, Leguminosae, Moraceae, Pinaceae, Platanaceae, Poaceae, Ranunculaceae, Salicaceae, Ulmaceae, Umbelliferae and Urticaceae. Pollen types with low occurrence frequency or those that were unidentifiable (including pollen fragments) were only recorded by concentration. The pollen monthly distribution for Fuzhou is presented in Table 1.

**Table 1:** Monthly distribution of pollen in Fuzhou.

Pollen type	Jan	Feb	Mar	Apr	May	Jun	Jul	Aug	Sep	Oct	Nov	Dec
Amaranthaceae						+				+	+	
Betulaceae			+	+	+	+				+		
Casuarinaceae			+	+	+							
Compositae						+	+	+	+	+	+	
Cupressaceae		+	+									
Leguminosae						+	+	+	+			
Moraceae		+	+	+	+	+			+			
Platanaceae			+	+								
Pinaceae		+	+	+	+				+	+		
Poaceae					+	+	+	+	+	+	+	
Ranunculaceae										+	+	
Salicaceae		+	+	+	+	+						
Ulmaceae			+	+								
Umbelliferae									+	+		
Urticaceae			+		+				+	+		
Other^*^	+	+	+	+	+	+	+	+	+	+	+	+

Other^*^, Pollen at low concentrations or that cannot be identified, as well as pollen fragments.

The “+” symbol indicates that the pollen type was detected during monitoring in Fuzhou in that month.

Statistical analysis revealed that the five main recorded types of pollen in Fuzhou were Moraceae, Urticaceae, Pinaceae, Cupressaceae, and Poaceae, which collectively accounted for 66.5% of the total pollen concentration collected during the study period.

As detailed in Table 2, Moraceae emerged as the predominant pollen type in Fuzhou, accounting for 40.7% of the total pollen count with a substantial concentration of 17,525 grains/m3. Urticaceae (12.1%) and Pinaceae (8.3%) were the second and third most main types, respectively. A notable year-on-year decline was observed in the total pollen concentrations of all major types. Moraceae demonstrated the most dramatic reduction, falling from 14,008 grains/m3 in the first year to 3,517 grains/m3 in the second. From a temporal perspective, the release of these main pollen types exhibited clear sequential patterns, with partial overlap in the dispersal periods of different families. Urticaceae, and Poaceae initiated their seasons in early autumn, whereas Moraceae, Pinaceae and Cupressaceae began in early spring. Peak concentrations for tree pollen types (Moraceae, Pinaceae, Cupressaceae) occurred in spring (March), while Urticaceae and Poaceae peaked in autumn (September and October, respectively). The highest single-day recorded concentration was for Moraceae on March 29, 2024 (1,489 grains/m3).

**Table 2:** Pollen data for main recorded types of pollen in Fuzhou.

Main pollen types	Annual pollen integral (APIn) (year1; year2)	% over total pollen grains	Date of peak day (grain/m^3^)^a^
Moraceae	17,525 (14,008; 3,517)	40.7	29/3/2024 (1,489)
Urticaceae	5,209 (4,298; 911)	12.1	25/9/2023 (335)
Pinaceae	3,572 (2,335; 1,237)	8.3	19/3/2024 (202)
Cupressaceae	1,480 (906; 574)	3.4	8/3/2024 (129)
Poaceae	821 (704; 117)	1.9	13/10/2023 (92)

^a^Value of peak day in grain/m^3^.

### Correlation Analysis for pollen concentration and baidu keywords

According to Spearman's correlation analysis (Figures 2C, D), Fuzhou pollen concentration showed a significant positive correlation with the Baidu Index keyword “pollen allergy” (*r* = 0.649, *p* < 0.001), indicating strong consistency between the two sets of pollen data. However, the correlation between pollen concentration and “allergic rhinitis” did not reach statistical significance (*r* = 0.242, *p* = 0.244).

### Daily outpatient visits for allergic rhinitis

**Figure 5:**
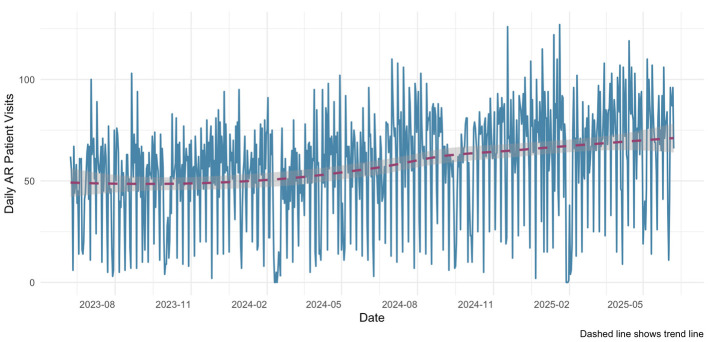
Time series of daily visits for allergic rhinitis from June 2023 to June 2025.

Based on a two-year analysis of daily outpatient visits for allergic rhinitis, this study preliminarily outlines the epidemiological characteristics of AR visits in Fuzhou. During the observation period, the total number of AR visits reached 42,166, with a daily average of 58 cases, indicating a gradual increasing trend over time (Figure 5). In terms of temporal distribution, patient visits exhibited densely clustered broad peaks with minor fluctuations, yet no distinct seasonal pattern was observed. Additionally, correlation analysis revealed no statistically significant association between daily AR visits and daily pollen concentration levels (*r* = −0.071, *p* = 0.056).

## Discussion

The increasing incidence of allergic diseases has become a focal point of public concern. Internet big data, with its near population-wide coverage, offers a valuable proxy for gauging public attention and interest in specific health issues—particularly in settings where large-scale field epidemiological data are lacking. The deterministic nature of IP address allocation further enhances the utility of search engine data for regional epidemiological studies, enabling highly precise geographic localization (30, 31). As the most widely used search engine in mainland China, Baidu provides a robust platform for such analyses. In this study, we first utilized the Baidu Index to understand real-world public attention toward pollen allergy in the Fuzhou area. These findings contribute to a deeper understanding of the epidemiological characteristics of allergic conditions in this region and offer meaningful references for designing targeted interventions.

During the study period, public attention to pollen allergy remained consistently high. Analysis of monthly trends revealed two annual peaks in search volumes for “pollen allergy” in both Fuzhou and nationwide, aligning with the characteristic seasonal dispersal patterns of pollen. The correlation between search trends for “pollen allergy” and “allergic rhinitis” was pronounced at the national level, likely attributable to the dominant role of pollen as a potent allergen in northern regions, where spring and autumn pollen concentration peaks closely synchronize with the onset of clinical symptoms (32, 33). In contrast, search volumes for “allergic rhinitis” in Fuzhou exhibited a sustained broad peak from early spring to late autumn, illustrating a more perennial pattern. This is likely attributable to Fuzhou's subtropical climate, which supports not only diverse botanical communities with overlapping pollen seasons but also promotes the year-round prevalence of indoor allergens such as dust mites, cockroaches, and mold (34–36).

Studies have shown that most patients with inhalant allergies exhibit polysensitization. A study on allergy patterns based on 7,148 allergic rhinitis patients in China revealed that 24.7% were sensitized to two allergens, and as many as 58.4% were sensitized to three or more allergens (37). Overlapping periods of exposure to multiple aeroallergens can exacerbate symptoms, particularly in warmer regions, because of the prolonged exposure to pollen allergens over several months (38, 39). In Fuzhou, this ecological context of coexisting multiple allergens contributes to an almost year-round incidence of allergic symptoms, distinguishing it from the predominantly seasonal pollen-driven pattern seen in higher latitude areas (40–42). Therefore, the synergistic effect and symptom intensification due to concurrent allergen exposure collectively constitute a composite exposure profile of allergic diseases.

Based on the two-year airborne pollen monitoring conducted in Fuzhou urban areas, the temporal pattern of pollen concentration was found to be consistent with the keyword “pollen allergy,” both showing two annual peaks in March–April and September–October. Specifically, significant discrepancies have been identified between the pollen concentrations and dispersal patterns observed in this region and those documented by scholars in other parts of China. These differences manifest in the timing of pollen dispersal, concentrations, and pollen types. Studies have indicated that pollen seasons tend to lengthen with increasing northern latitude (33). Northern China experiences two annual pollen peaks: a spring peak (March–May) dominated by tree pollen (e.g., Cupressaceae, Ulmaceae), and a summer/autumn peak (August–September) characterized primarily by herbaceous pollen, notably from *Artemisia, Humulus*, and Amaranthaceae (43). The temperate monsoon climate of northern China, favors high pollen-yielding plants such as poplar, elm, pine, and cypress, which are the primary cause of spring pollen allergies (5). In autumn, pollen from mugwort predominates, especially in the northwest where it is extensively planted for desert control, making *Artemisia* exposure a major allergen in this region (15). Our observations in Fuzhou, located in southern China, revealed that the duration of its two annual pollen peaks was shorter than that documented in northern regions (42). In Fuzhou, the two peaks occur in March–April and September–October, while the onset of the spring pollen season is relatively similar between Fuzhou and northern China, though its duration is shorter; the autumn peak in Fuzhou occurs slightly later than in the north, and the pollen interval between the two peaks is notably longer in Fuzhou. Consistent with patterns observed in many southern China areas, Fuzhou pollen concentrations in spring are significantly higher than in autumn, and the main airborne pollen originates primarily from local trees and urban greenery used in park landscaping (44).

Among the major tree pollen types in Fuzhou, the structural and morphological characteristics of key species contribute significantly to the observed spring peak. Mulberry pollen grain characteristically small, light, and is produced in large quantities due to the inflorescence morphology of the trees, leading to substantial atmospheric release. Pine pollen possesses a unique vesiculate structure that significantly enhances its buoyancy, enabling it to disperse over higher altitudes and longer distances while remaining airborne for more than 12 h (45). Cupressaceae plants produce substantial amounts of pollen during their concentrated spring pollination period (46), their pollen grains are small and prone to rupture, which facilitates the release of allergenic proteins and significantly increases allergy risks in the surrounding environment (47). Multiple studies have demonstrated that these pollen were major trigger of spring allergic rhinitis in many regions, and its allergenic proteins have been clearly identified (46, 48–51). The spring search peak observed in this study aligns with the local pollen dispersal period, further supporting our inference that public online attention to “pollen allergy” is associated with seasonal exposure to major airborne allergenic pollen in the environment.

During the summer and autumn seasons, the main pollen sources shift to Urticaceae and Poaceae. Urticaceae pollen grains are small (approximately 10 μm in diameter) and possess strong allergenic potential (52). Parietaria, which are widely distributed as common weeds in the Mediterranean region, produce pollen that represents a major cause of allergies in the area, affecting about 25–30% of allergic patients locally (53). Weed species, known for their robust vitality and strong environmental adaptability, thrive in urban green belts and roadside areas, allowing pollen from weeds such as those in the Urticaceae family to become one of the dominant pollen types. Furthermore, the relatively compact central urban area of Fuzhou is surrounded by farmland ecosystems that cultivate crops such as corn and rice, which contribute to a baseline concentration of Poaceae pollen. This is compounded by the presence of common urban grasses like *Poa annua* L. and *Setaria viridis* (L.) P.Beauv. within the city. Most Poaceae species are annual or perennial grasses characterized by hollow stems. As wind-pollinated plants, many possess pendulous anthers and produce substantial quantities of morphologically similar pollen grains, which are released into the atmosphere and are now recognized as one of the major airborne allergens (54). During the pollen season, numerous grass species shed highly concentrated pollen, triggering a range of allergic symptoms in sensitized individuals—from seasonal rhinoconjunctivitis to bronchial asthma (55). The combination of agricultural and urban sources results in a higher concentration and longer dispersal period of grass pollen. This interactive pattern of pollen production is particularly pronounced in the spatially constrained central urban area of Fuzhou. It also reflects the complex relationship between natural vegetation and human-made ecosystems in the context of rapid urbanization.

Subsequently, we also considered the reasons for the variation in pollen concentration over the two years. Pollen dispersal are directly influenced by vegetation distribution, which itself is primarily determined by climate, geographical conditions, and the plants' inherent adaptability (56). Various climatic factors, such as rainfall, humidity, wind speed and direction, temperature, and hours of sunshine (57, 58), and air pollutants (59), exert both indirect and direct effects on airborne allergen. Given the relatively brief duration of this pollen monitoring period, the observed temporal variations in the pollen season are likely to be influenced more significantly by the combined effects of short-term meteorological conditions, pollutants or human factors. A logical next step is, therefore, to investigate the specific allergenic potential of these dominant local pollen types to accurately assess their public health burden.

Over time, the composition, concentration, and seasonal dynamics of airborne pollen undergo significant shifts. Some scholars have suggested that climate change has accelerated the onset of flowering periods, increased pollen production, and extended pollen seasons; extreme weather events and air pollution have further exacerbated pollen yields and sensitivities, and urbanization has heightened tree pollen levels, leading to spring peak pollen concentrations gradually surpassing summer/autumn levels (43). A long-term study in Brussels, for instance, revealed divergent trends over four decades: most tree pollen types increased, while most grass pollen types decreased (60). In Fuzhou, an earlier survey conducted in 1987 using the gravimetric method reported a major pollen peak in March and a minor one between November and December, with Pinaceae, Cannabis, and Poaceae as the main types.

Our current monitoring reveals notable changes both in the timing of pollen peaks and in the spectrum of dominant pollen taxa. These shifts likely reflect broader environmental transformations over the past four decades—such as alterations in local vegetation, air-quality patterns, and Fuzhou's accelerated urbanization—as well as regional climatic trends including changes in seasonal precipitation and cumulative solar radiation (60).

Then, differences in sampling methodologies may also contribute to the observed discrepancies. Gravitational sampling relies entirely on the natural settling of pollen under gravity, which results in lower collection efficiency for smaller pollen grains compared to active volumetric methods. In contrast, the Burkard volumetric spore trap—used in the current study—demonstrates higher sampling efficiency, capturing over 80% of particles smaller than 10 micrometers (17). Pollen grains from Moraceae and Urticaceae, which ranked first and second in dominance in our study, generally range between 10–18 μm in diameter. These are considered small pollen grains, for which the Burkard sampler achieves high collection efficiency, likely yielding more accurate monitoring data.

Spring-blooming plants are generally taller than those flowering in summer and autumn. A sampler placed at a height of around 20 meters may thus capture spring pollen more effectively (61). In addition, variations in inflorescence structure among spring-blooming species can lead to significant differences in pollen release. For instance, species such as mulberry bear catkins—dense, hanging clusters of male flowers that release enormous quantities of pollen. This may partly explain why pollen concentrations in spring are markedly higher than those in autumn.

Furthermore, the significant positive correlation between pollen concentration and searches for “pollen allergy” demonstrated that online public attention closely reflected actual airborne pollen levels in Fuzhou, supporting the utility of Baidu Index data for monitoring population-level allergic responses. In contrast, no significant correlation was observed between pollen concentration and searches for “allergic rhinitis.” This may be attributed to the broader scope of the term, which encompasses various triggers beyond pollen (e.g., dust mites and mold), as well as the influence of factors such as medication adherence on healthcare-seeking behavior. Furthermore, the allergenic risk posed by pollen in Fuzhou warrants further investigation.

Additionally, we extracted two years of outpatient data on allergic rhinitis consultations to conduct a preliminary investigation into potential effects under pollen exposure. According to some studies, high levels of airborne pollen appear to correlate positively with allergic rhinitis and asthma responses in temperate climates (57, 62). However, this correlation is not always evident (63). During the monitoring period of this study, the total number of allergic rhinitis visits in Fuzhou showed a gradual increasing trend, which may be attributed to improved public awareness of the disease, rising diagnosis rates, or long-term changes in another environmental factor. Meteorological conditions may still play a significant role: while rainfall can reduce airborne pollen concentrations, it may simultaneously deter patients from seeking medical care, leading to a decline in recorded visits. Conversely, sunny and windy conditions facilitate pollen dispersion while encouraging outdoor activities, thereby increasing both exposure and the risk of symptom onset; at the same time, extensive research indicates that meteorological conditions can also directly impact health (64). This “negative” finding is of considerable implication, suggesting that the drivers of AR-related healthcare-seeking behavior are far more complex than simple immediate pollen exposure.

This study demonstrates four key strengths: (1) Given the lack of long-term epidemiological data, online search data (Baidu Index) provided a viable alternative for the efficient and cost-effective analysis of disease trends. (2) By combining pollen monitoring with internet search behavior data (Baidu Index), this study provides a more comprehensive understanding of pollen exposure and its relationship with public health responses. (3) The use of Burkard volumetric sampler enabled continuous pollen collection over two full years, offering daily concentration data with high temporal resolution and minimizing operational bias associated with discontinuous methods. (4) This study provides a foundational dataset for subsequent research into longer-term pollen variations and pollen-meteorology studies, thereby facilitating further advancement in local pollen allergy research.

This study has several limitations. First, the Baidu search engine does not allow keywords to be restricted to specific categories or subcategories (like specific types of pollen – “Moraceae pollen allergy”), which may have affected the comprehensiveness of search trend analysis. Second, the geographical scope was limited to Fuzhou city without distinguishing between urban and rural users, potentially overlooking spatial heterogeneity in pollen exposure and search behavior. Third, the user base of Baidu is predominantly composed of middle-aged and young individuals, which may introduce age-related bias. Additionally, this study included monitoring data from only one site over a two-year period, making it difficult to distinguish long-term trends from short-term fluctuations. The lack of individual-level exposure and symptom data may also lead to an underestimation of the complex interactions between environmental and meteorological factors.

Hence, the findings should be interpreted with caution, and future studies incorporating multi-source data, longer time spans, and individual-level information are warranted to validate and extend these observations.

## Conclusions

In summary, this study provides a timely analysis of airborne pollen patterns and public awareness in a subtropical urban setting by combining digital epidemiology with traditional monitoring. The findings offer a scientific basis for the clinical management of pollen allergies and lay the groundwork for forecasting pollen trends under the influence of climate change and rapid urbanization. This research serves as a critical step toward developing effective public health strategies and early warning systems to mitigate the impact of pollen-related allergic diseases in evolving urban environments.

## Data Availability

The original contributions presented in the study are included in the article/supplementary material, further inquiries can be directed to the corresponding author.
